# Longitudinal study of association between quality of life and grit in internal medicine residents in Ramathibodi Hospital

**DOI:** 10.1186/s12909-024-06011-y

**Published:** 2024-09-30

**Authors:** Pattarapol Kitthavorn, Sahaphume Srisuma, Sirichai Hongsanguansri, Thotsaporn Morasert

**Affiliations:** 1 Department of Medicine, Faculty of Medicine Ramathibodi Hospital, Mahidol University, Bangkok, Thailand; 2 Division of Clinical Pharmacology and Toxicology, Department of Medicine, Faculty of Medicine Ramathibodi Hospital, Mahidol University, 270 Rama 6 Road, Toong Phaya Thai, Ratchathewi, Bangkok, 10400 Thailand; 3 Department of Psychiatry, Faculty of Medicine Ramathibodi Hospital, Mahidol University, Bangkok, Thailand; 4 Department of Medicine, Suratthani Hospital, Surat Thani, Thailand

**Keywords:** Grit, Resident, Quality of life, Stress, Learning, Training

## Abstract

**Background:**

Grit refers to a combination of perseverance and passion, which is essential for long-term success. Grit is associated with higher education attainment and a lower likelihood of burnout. Understanding how grit evolves during internal medicine residency training is important for providing better support to residents. The primary objective of the current study was to evaluate potential changes in the level of grit throughout the training program and investigate the association between grit and stress levels, quality of life (QoL), and satisfaction of learning scores.

**Methods:**

We conducted a prospective study involving internal medicine residents enrolled in training programs from July 2022 to July 2023, except for the second-third-year residents of dermatology and neurology programs and the third-year residents of haematology and oncology programs, which are not part of the same training regimen. Data collection was conducted using questionnaires at the beginning of the training year and every three months until the end. Collected data included age, gender, habitat, working experience, financial burden, marital status, rotations, grit scores, QoL, stress score, and satisfaction of learning score during each trimester. Changes in grit over the year were examined using repeated measures analysis of variance adjusted by training program, financial status, working experience, habitat, marital status, and age. We performed multivariable linear regression models to explore associations between grit and the following domains: QoL, ST-5, and SoLs.

**Results:**

Of 122 internal medicine residents in all three years of training, one resident dropped out during training, and another declined to participate. A total of 120 residents were included. Grit did not significantly change over time. Subgroup analysis of the first-year resident group revealed that grit significantly decreased (*P* = 0.049). Multivariable analysis revealed that grit was positively associated with QoL (β = 0.01, *P* < 0.001) and satisfaction of learning score (β = 0.07, *P* < 0.001) but inversely associated with stress score (β = − 0.04, *P* < 0.001).

**Conclusions:**

Of all residents, grit did not change over a year. However, only the first-year residents reported a decrease in grit during a training year. Throughout first-year residents’ internal medicine residency training, grit was positively associated with QoL and satisfaction of learning score but negatively associated with stress level. These findings suggest the need for targeted interventions to support first-year residents and enhance their resilience throughout training.

**Supplementary Information:**

The online version contains supplementary material available at 10.1186/s12909-024-06011-y.

## Introduction

Grit, characterized by perseverance and passion for long-term goals, has emerged as a pivotal predictor of success across diverse realms, including career trajectory [1]. Grit has been reported to play a greater role than talent alone in determining a person’s ability to achieve enduring objectives and is particularly important as individuals navigate the demands of challenging professions. Higher levels of grit are correlated with older age, advanced education, better self-control, greater conscientiousness, and fewer career changes. Like intelligence quotient and education level, grit has been studied in terms of its association with long-term success [1–4].

In healthcare settings, the significance of grit has prompted the exploration of interventions for enhancing resident physicians’ resilience and long-term success. Recent studies of medical students revealed positive associations between grit and medical professionalism and between grit and academic performance, as well as an inverse relationship between grit and stress levels [5, 6]. These findings emphasize the relevance of grit in shaping professional behaviour and academic achievement.

The demanding nature of work environments, including medical residency training programs, may influence individual grit levels. Recent studies in resident physicians revealed a positive association between grit and the well-being of residents and an inverse association with emotional exhaustion [7]. Knowing whether there are changes in grit, quality of life (QoL), stress, and learning satisfaction during training or if there are any factors associated with them would be beneficial for setting up residency training that enhances both grit and QoL.

The primary objective was to determine the association between grit, QoL, stress level, and learning satisfaction of internal medicine residents during an academic year. The secondary objectives were (1) to determine how grit, QoL, stress level, and learning satisfaction of internal medicine residents were changed during a training year, and (2) to identify factors associated with grit, QoL, stress level, and learning satisfaction among resident physicians.

## Methods

### Study setting

The current study was a prospective longitudinal study conducted at the Department of Internal Medicine, Faculty of Medicine, Ramathibodi Hospital, Mahidol University, Bangkok, Thailand. The participants were internal medicine resident physicians (first- to third-year residents). The fast-track residents trained under the same regimen as internal medicine residents included first-year residents of neurology and dermatology programs and the first-and second-year residents of oncology programs. The second, and third-year residents of neurology, haematology and dermatology programs and third-year residents of oncology were excluded from the current study because of the different training regimens from those of internal medicine residents. The summary of inclusion subject were show in Table 1. The data collection period was one training year from 1 July 2022 to 30 June 2023.

The Ramathibodi Internal Medicine residency training program has one main training program (RAMA-RAMA) and 3 co-training programs with other tertiary care hospitals in different regions of Thailand. All residents have rotations in Ramathibodi Hospital, a university hospital or so-called super-tertiary hospital. Residents also have rotations to other tertiary care hospitals, which have higher numbers but less complexity of the cases, compared to Ramathibodi Hospital. Residents in the main program (RAMA-RAMA) have rotations in a new Ramathibodi campus (Chakri Naruebodindra Medical Institute; CNMI). Residents in the three co-training programs will have rotations to one of the three tertiary hospitals: Suratthani Hospital (SRT, in the southern region), Buriram Hospital (BRM in the north-eastern region), and Sawanpracharak Hospital (SPR, in the central region outside Bangkok). Rotations of residents were described in Supplement 1.

This study is approved by the Institutional Review Board of the Faculty of Medicine at Ramathibodi Hospital, Mahidol University (MURA2024/225). The responses of participants were voluntary. There is no incentive for the participants. The details and objectives of the study were presented, and informed consent was obtained electronically using Google Forms.

**Table 1:** Residents subject included in study

	1st year	2nd year	3rd year
General medicine (Main program and all 3 co-training programs)	Included	Included	Included
Oncology program	Included	Included	Not Included
Neurology program	Not Included	Not Included	Not Included
Dermatology program	Not Included	Not Included	Not Included
haematology program	Not Included	Not Included	Not Included

### Study tools

The data were collected using participant-administered online questionnaires via Google Forms. All questions for basic characteristics and scoring were required to complete the questionnaire. Each participant was asked to complete the questionnaire at the beginning of the year of training, then every 3 months, to evaluate the change and progression of grit and other factors during each trimester. Thus, each participant answered the questionnaire five times. The questionnaires were distributed on the first Tuesday of the academic year, and on the last Tuesday of the 3rd, 6th, 9th, and 12th months (end of the training year). Responses were acquired within 2 weeks after being distributed. The reminder e-mails were sent to the participants who had not completed the questionnaires on day 7, 10, and 14 after first distribution. The collected data were as follows:

- Baseline characteristics included age, gender, year of working experience, financial status (collected as a self-perceived financial burden and non-financial burden), marital status (single, in relationship, and married), habitat (private residence or hospital dormitory), and training program (fast-track program or co-training program).
- Rotation: over the year, each resident will go through 13 rotations. Each rotation lasts for 4 weeks. We collected data during three rotations per trimester to investigate the effect of demanding work on grit. The list of rotations was as follows:
  - Five intensive care units: Intensive ward 1, intensive ward 2, Cardiac intensive unit (CICU), Intermediate ward.
  - Three general inpatient departments general male IPD, general female IPD, observe ward).
  - Two subspecialty units: haematology-oncology ward and stroke unit.
  - Outpatient department rotation (OPD).
  - Emergency room rotation (ER).
  - Vacation and elective rotation.
  - Subspecialty rotations included cardiology, gastrointestinology (GI), pulmonology, nephrology, infectious disease (ID), rheumatology, dermatology, oncology, haematology, endocrinology, and neurology.
  - Rotation to other hospitals (CNMI, SRT, SPR, BRM).
- Grit scores were collected using questions from the Grit questionnaire developed by Duckworth (2007), which were translated into Thai and validated by Jongjumruspun M. The questionnaire consisted of 12 questions. Grit scores were reported using a rating scale ranging from 0 to 5. Higher scores indicate a higher level of grit. Permission to use the questionnaire was obtained. Cronbach’s alpha of grit score was previously reported as 0.71 [8].
- QoL data were collected using the World Health Organization Quality of Life Brief Version, translated into Thai and validated by Suanprung Psychiatric Hospital (WHOQOL – BREF –THAI). The questionnaire comprised 26 questions from four domains: physical, psychological, social relationship, and environment. Permission to use the questionnaire was obtained. Cronbach’s alpha of the WHOQOL-BREF, compared with the WHOQOL-100, was 0.8406 [9].
- The stress screening tool, evaluated using the Srithanya-5 (ST-5) score, consisted of five questions. Scores of 0–4 indicate low stress, scores of 5–7 indicate moderate stress, scores of 8–9 indicate high stress, and scores of 10–15 indicate very high stress. Permission to use the questionnaire was obtained. The Cronbach’s alpha of the ST-5 score was 0.85 [10].
- Satisfaction of learning score (SoLs) was defined as the participant’s self-satisfaction with their own learning capacity. Respondents answered using a self-perceived visual analogue scale with a rating scale from 0 to 10.
- Open-ended questions were included in an optional section for participants to express their opinions about the factors affecting their learning.

Each participant was assigned a unique ID to track the individual data for each person. Data were sorted and collected using the unique ID to maintain participants’ anonymity. Data were stored in a security database accessible only by the principal investigator.

### Data analysis

We used repeated measures analysis of variance (RM-ANOVA) to detect overall differences between multiple grit, QoL, ST-5 and SoLs measurements of the same resident (correlated samples) throughout the training period. We planned to perform subgroup analysis for each year of residency training. The RM-ANOVA was adjusted for various confounding covariates, including training program, financial status, years of working experience, living situation, marital status, and age. Statistical significance was determined at a threshold of *p* < 0.05, and corrections were applied using Box’s conservative epsilon.

The associations between grit and the following factors, including QoL, ST-5, and SoLs were separately examined using multivariable linear regression models. These models were adjusted for trimester, training program, financial status, years of working experience, living situation, marital status, and age.

Furthermore, multivariable linear regression models were applied to explore the potential factors (trimester, training program, financial status, working experience, living situation, marital status, age, and rotations) contributing to grit, QoL, ST-5 score, and SoLs changes. Analyzes were conducted separately for each year of residency because of differences in rotation types and training regimens. Coefficients (β) and 95% confidence intervals (CI) were reported, with statistical significance set at *p* < 0.05. If there was missing data, the data were analyzed using a complete case analysis.

The linear regression assumptions, including homoscedasticity, normality, and multicollinearity were checked using Breusch-Pagan hettest, Shapiro-Wilk W normality test, and Variance inflation factor, respectively.

## Results

Of 122 internal medicine residents from all 3 years of training, one resident dropped out during training, and another declined to participate. A total of 120 residents were included. Table 2 reported demographic data. Initial mean grit scores were 3.31, 2.96, and 3.09 for first-, second-, and third-year residents, respectively.

**Table 2:** Participants’ demographic data

Factors		First-year	Second-year	Third-year
		n, (%)	n, (%)	n, (%)
Total		50	37	33
Age, year, mean ± SD		27.3 ± 0.9	28.3 ± 0.9	29.9 ± 0.9
Gender	Male	21 (42)	21 (57)	20 (61)
	Female	29 (58)	16 (43)	13 (39)
Training program				
Internal medicine	RAMA-RAMA	22 (44)	21 (57)	21 (64)
	RAMA-SPR	6 (12)	5 (14)	6 (18)
	RAMA-SRT	6 (12)	6 (16)	6 (18)
	RAMA-BRM	2 (4)	2 (5)	0
Subspecialty program				
	Haematology	3 (6)	0	0
	Neurology	3 (6)	0	0
	Oncology	2 (4)	3 (8)	0
	Dermatology	6 (12)	0	0
Work experience, year	1–2	2 (6)	1 (3)	0
	3–4	45 (90)	25 (68)	8 (24)
	5–6	2 (4)	11 (30)	24 (72)
	9	0	0	1 (3)
Marital status	Single	30 (60)	15 (41)	7 (21)
	In relationship	18 (36)	22 (59)	23 (70)
	Married	2 (4)	0	3 (9)
Living situation	Hospital dormitory	27 (54)	22 (59)	19 (58)
	Private residence	23 (46)	15 (41)	14 (42)

**Fig. 1:**
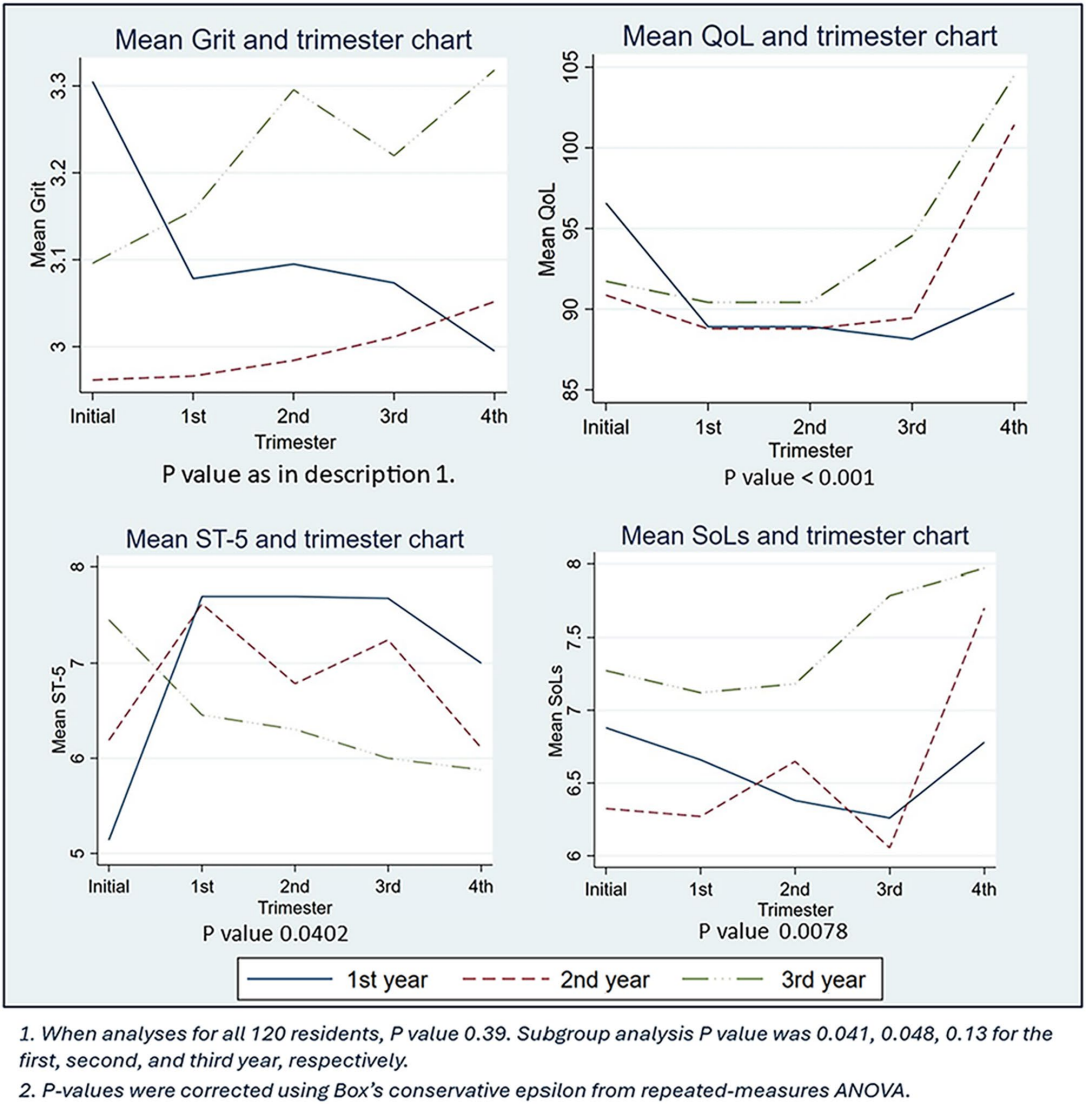
Mean Grit, QoL, ST-5, and SoLs and trimester chart

Grit did not significantly change over time when analyzed for all 120 residents. Subgroup analysis of first-year residents showed that grit significantly decreased in the training year). Of all 120 residents, SoLs significantly vary over time. QoL significantly decreased in the first trimester and increased in the third trimester. Among the first- and second-year residents, ST-5 scores significantly increase during the first trimester but decrease in the subsequent trimesters. Figure 1 shows the mean grit score, QoL, ST-5 score, and SoLs.

**Fig. 2:**
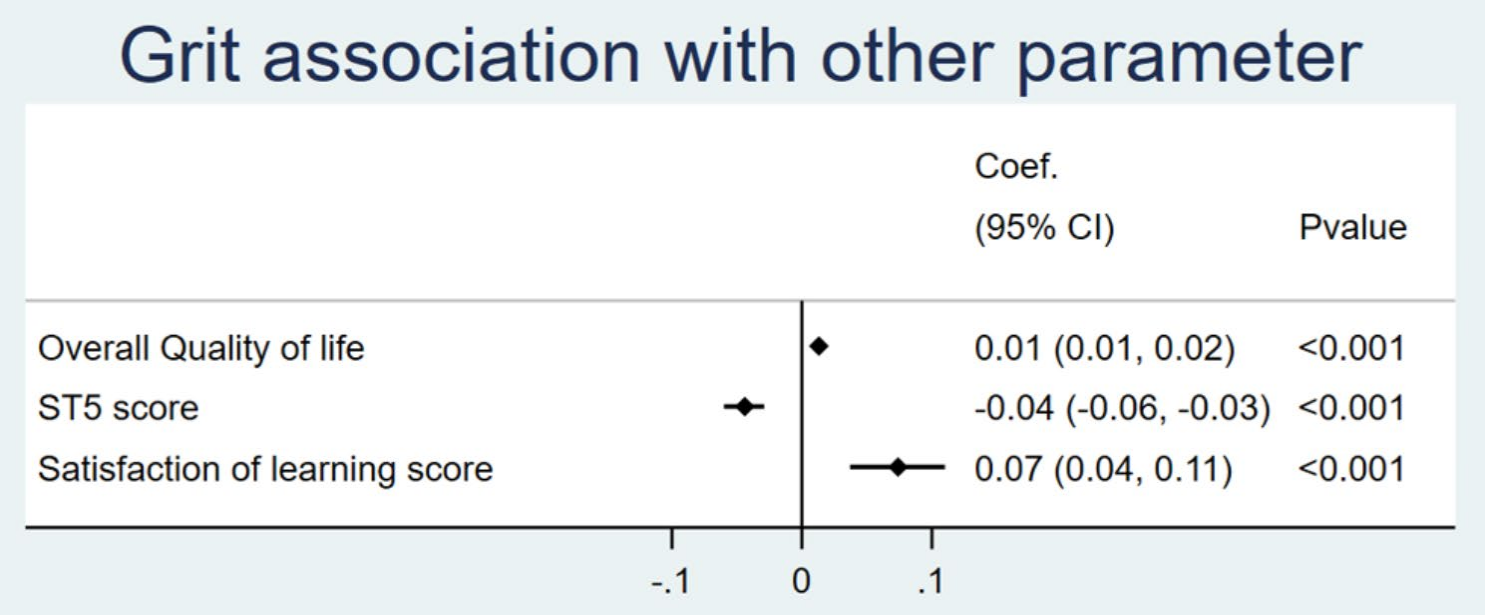
Forest plot of linear regression of factor associated with grit

Of all 120 residents, multivariable linear regression analysis with and adjusting for baseline characteristics (age, gender, training program, financial status, working experience, habitat, and marital status) revealed a positive association between grit and the following factors, including QoL and SoLs. Grit was inversely associated with ST-5 score (Fig. 2).

**Fig. 3:**
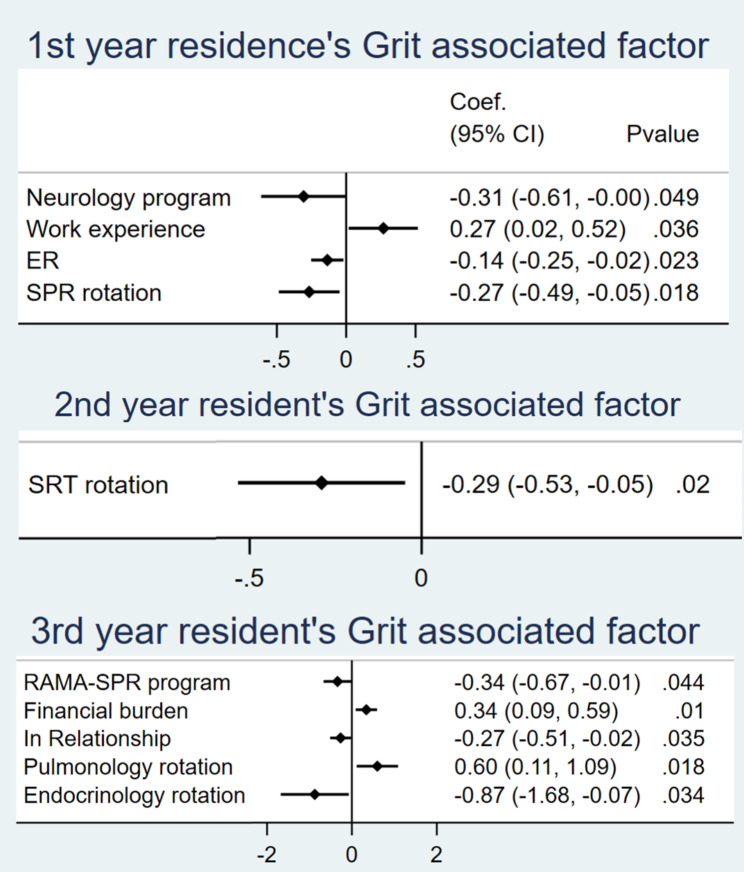
Grit-associated factors

**Fig. 4:**
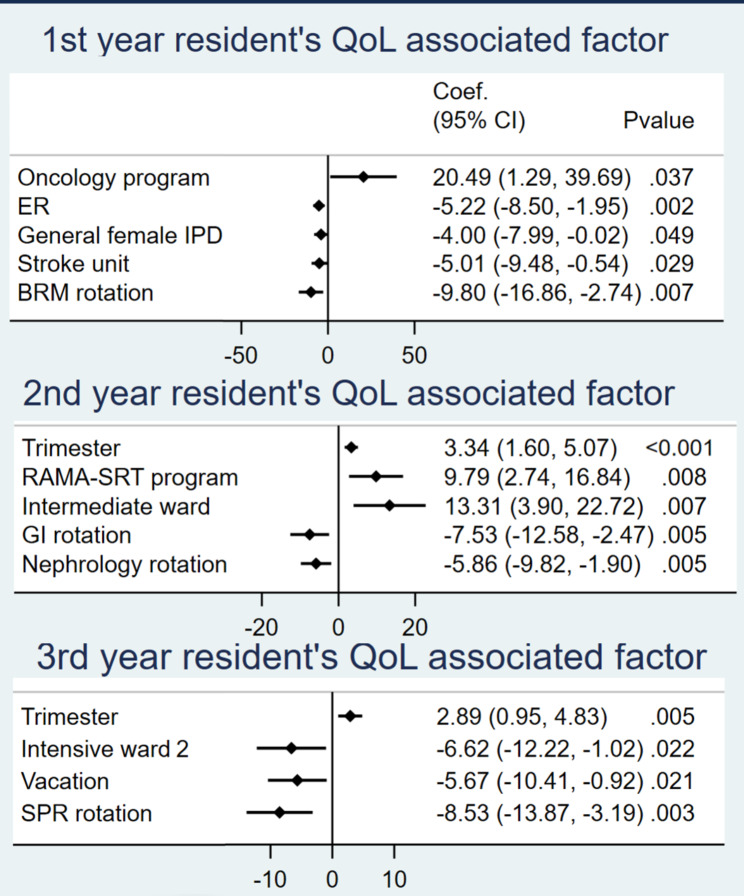
QoL-associated factors

**Fig. 5:**
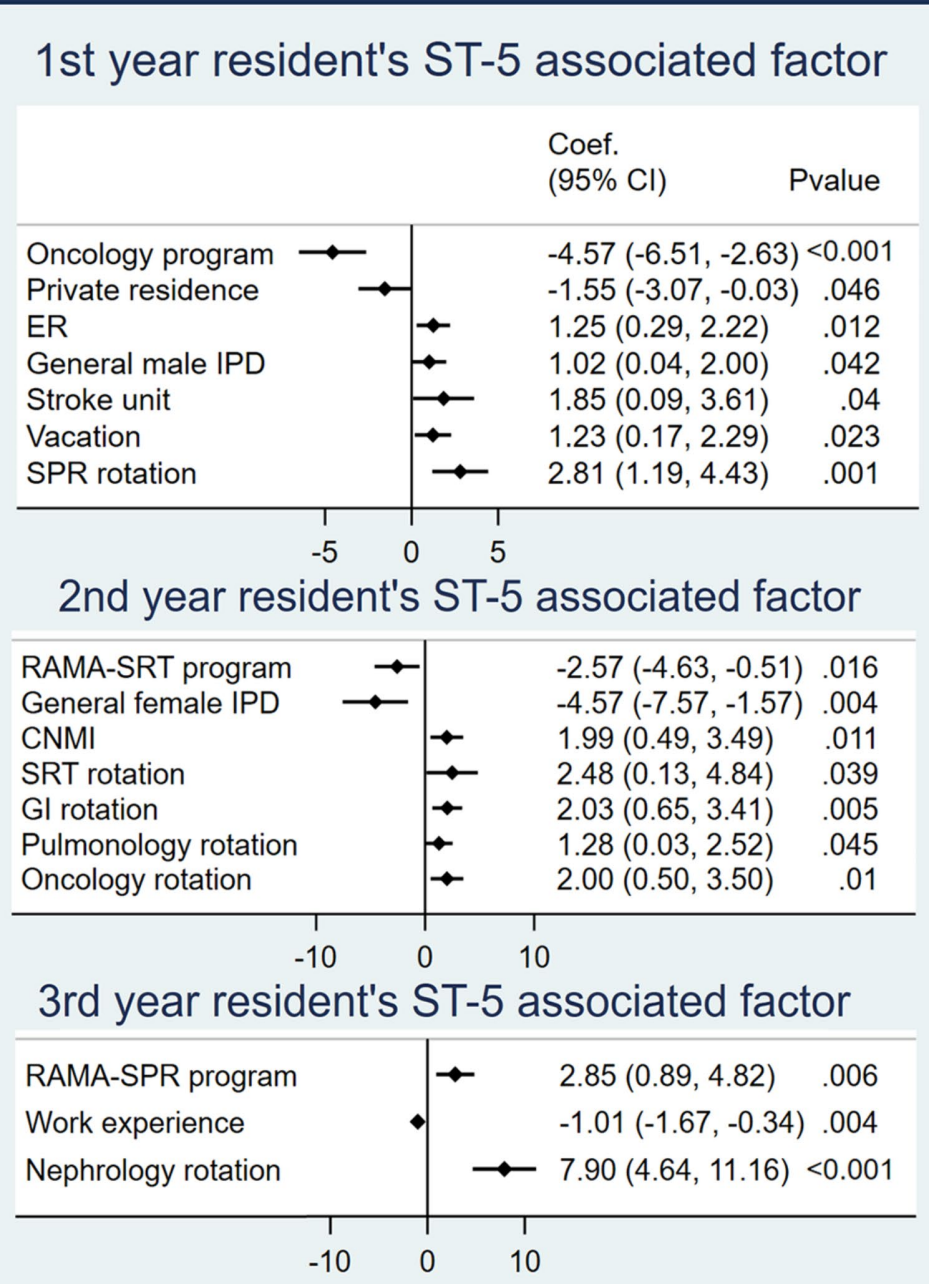
ST5-associated factors

**Fig. 6:**
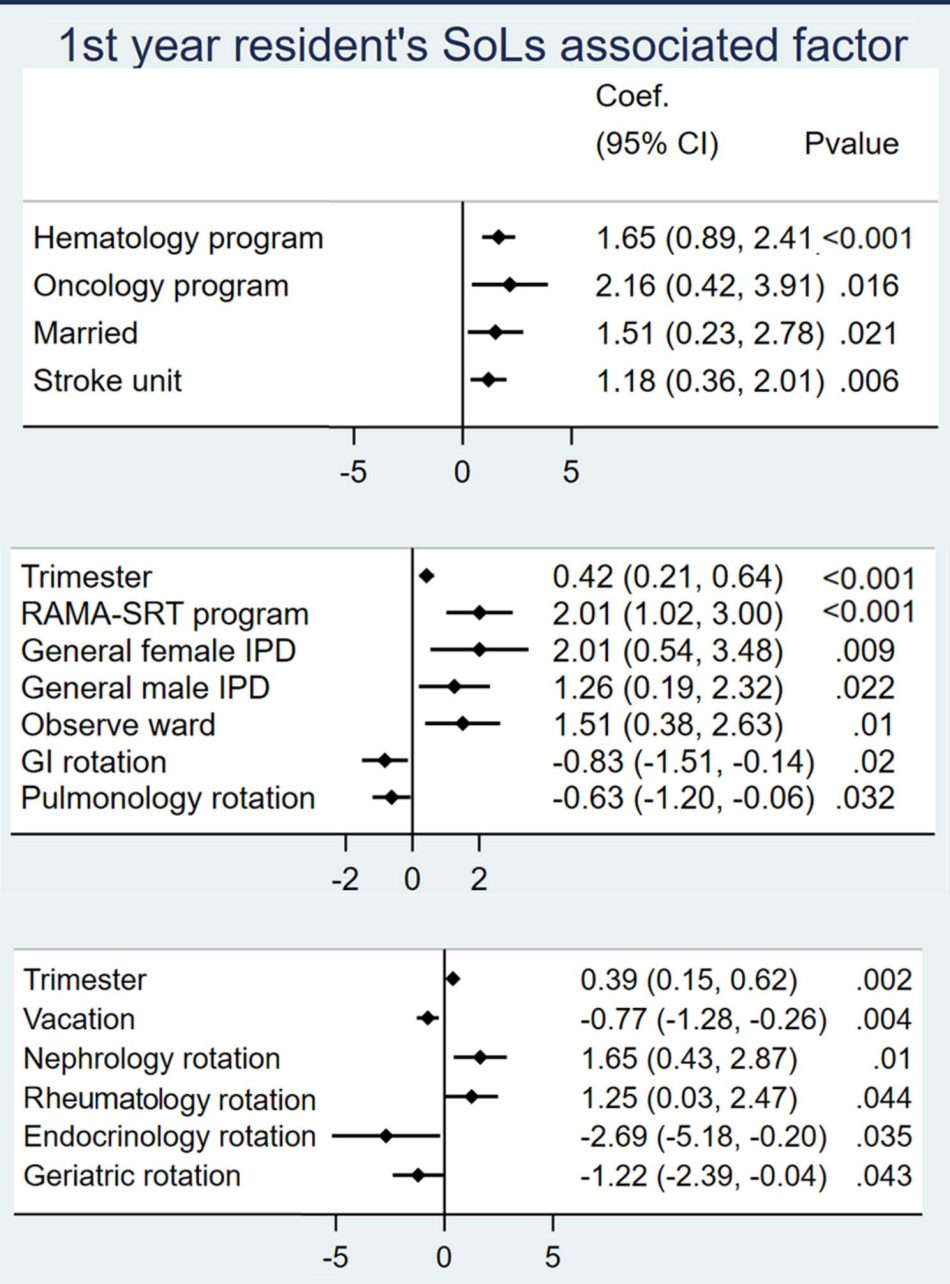
SoLs-associated factors

Factors significantly associated with grit, QoL, SoLs, and ST-5, as determined by multiple linear regression, are demonstrated in Figs. 3, 4, 5 and 6. The analysis controlled for baseline characteristics: age, gender, training program, financial status, working experience, habitat, marital status, and rotation. Forest plots with all factors of each analysis are provided in supplement data 2.

### First-year residents

Residents in the neurology program who rotated in internal medicine regimen for only one year of their whole training were associated with lower grit scores. More working experience was associated with higher grit scores. Grit scores were inversely associated with ER and SPR rotation, which were high workload rotations. Oncology residents had a positive association with higher QoL and lower ST5 scores. QoL was inversely associated with ER, general female ward, stroke unit and BRM rotation. ST-5 score was significantly lower in residents who lived in a private residence. A higher ST-5 score was associated with ER, general male ward, stroke unit, SPR rotation, and vacation. SoLs were higher in residents in the haematology and oncology programs, married residents, and residents in the stroke unit.

The stroke unit rotation was related to lower QoL and higher stress levels. However, it was also associated with higher SoLs. Anonymous responses from first-year residents revealed that, in the stroke unit rotation, students were required to work with a total of six patients without the chief resident. Neurology fellows and attending staff often ordered students to perform various tasks that were sometimes challenging for them as first-year residents. Some respondents reported these experiences as counterproductive and stressful, while others found them a good learning opportunity.

### Second-year residents

Grit was inversely associated with SRT rotation. QoL was positively associated with residents in the RAMA-SRT program, later trimesters, and the intermediate ward. QoL was significantly lower in the GI and nephrology rotations. ST-5 score was inversely associated with general female IPD rotation and residents in the RAMA-SRT program. ST-5 score was positively associated with CNMI, GI, pulmonology, and oncology rotation. SoLs were significantly higher in later trimesters, residents in the RAMA-SRT program, general female, general male, and observation ward. SoLs were significantly lower in GI and pulmonology rotation.

The GI and nephrology departments were associated with lower QoL in the second year. Residents reported that these rotations, which were very early in the morning, deprived them of sleep. Furthermore, residents in the nephrology rotation reported feeling exhausted due to the workload.

### Third-year residents

Grit was significantly higher in residents experiencing financial burdens, and those in the pulmonology rotation. Grit was significantly lower in residents in the RAMA-SPR program, in relationships, and during the endocrinology rotation. QoL was positively associated with later trimesters. QoL was inversely associated with Intensive ward, vacation, and SPR rotation. ST-5 score was positively associated with the RAMA-SPR program and nephrology rotation. ST-5 score was negatively associated with working experience. SoLs were higher in nephrology and rheumatology rotations and later trimesters. SoLs were lower in vacation, endocrinology, and geriatric rotation. Third-year residents in a relationship stated that they spent more time with their partners and focused on post-residency training plans rather than pursuing their original career goals.

## Discussion

This study reported changes in grit, QoL, stress, and satisfaction of learning in an internal medicine residency program, and factors associated with them. The results showed that grit was positively correlated with QoL and SoLs, while inversely correlated with stress. The current results revealed a significant decline in grit over the training year among first-year residents. Although the results for overall residents were not statistically significant and differed between subjects each year, the trend in grit among second-year residents was steady throughout the year. It increased gradually over the year among third-year residents. The results revealed that working experience was associated with higher grit and lower ST-5 scores. These findings suggest that residents’ adaptiveness to work-related difficulties improved with age or experience. Thus, this may also suggest that reduced grit in the first year of training could be recovered in subsequent years of training.

Consistent with previous research, grit was positively associated with QoL [7] and inversely associated with stress levels [10]. Self-perceived satisfaction also increased with grit, implying that individuals with higher grit levels were more satisfied with their learning capabilities. Regarding factors associated with grit, the results revealed that third-year residents in relationships exhibited lower grit scores, possibly indicating that being in a relationship negatively influenced their original passions and goals. Time and focus are required for both residency training and maintaining personal relationships. Poor work-life balance may be related to stress, relationship issues and diminish residents’ wellness [11].

The financial burden was associated with a higher level of grit among third-year residents, possibly because of better financial behaviour. This finding is consistent with a prior report that individuals with higher grit levels have better financial behaviour and prioritize education-related spending [12].

Specific rotations, such as ER, SRT, and SPR rotations, were associated with lower grit and higher stress. This finding is consistent with the demanding nature of this type of work, which may mentally and physically exhaust residents [13, 14]. The training program should consider adjusting workload in rotation with higher stress to maintaining residents’ QoL, and grit [15].

Additionally, residents who lived in their own residences exhibited lower stress levels compared with those living in dormitories. This finding is in accord with a previous report that dormitory living can lead to feelings of incompatibility, loneliness, discomfort, and anxiety [16]. This finding suggests the need for further studies to explore ways of improving dormitory accommodation to lessen residents’ stress.

Counterintuitively, vacation was associated with higher stress and lower QoL. This finding is potentially attributable to comparison bias. Because completing the questionnaire takes time, participants may have chosen to complete the questionnaire upon returning from vacation or resuming work. This might have led residents to compare their previous leisure time and the current work period. A study by Bloom J et al. reported that vacation improved health and well-being during the vacation, but the benefits faded out in the first week of work resumption. Types of activity during vacation may be related to difference in benefit of vacation [17]. Having work-related activities during vacation, which may happen in some residents, reported to have weak relationships with employees’ recovery and well-being. Further study on vacation activity and effect of vacation on grit, stress, and QoL of resident would be benefit for enhancing benefits of vacation [18].

Subspecialty rotation in third-year residents showed both negative and positive associations with grit. It is essential to consider the distinct nature of third-year subspecialty rotations as elective rotations that are typically less demanding than other rotations. However, third-year residents often choose elective rotations based on their aspiring fellowship programs and might be stressed about the desire to leave a positive impression on subspecialty staff. The limited number of third-year residents in the elective departments (1–3 people in each department during the whole year) may have also introduced inaccuracies by not fully capturing the effects in the linear regression model. Notably, non-subspecialty rotations among second-year residents, which only occurred in the final rotation of the training year, were associated with higher levels of grit, greater SoLs, and lower stress levels. This effect may be attributed to the timing of the final questionnaire, administered 1 month after the board examination, potentially reflecting diminished stress and enhanced self-confidence following exam success.

### Limitations

Determining the threshold for classifying individuals as “gritty” on the basis of their grit scores is challenging. In the previous research on grit in medical student in Ramathibodi hospital and using the same grit measurement questionnaire, the mean grit were 3.3. It is difficult to quantify the extent to which each factor influences grit precisely. Moreover, the timing of questionnaire responses may have impacted the validity of outcomes because participants might respond to questionnaires when they have free time, leading to comparisons between moments of relaxation and previous rotations. Additionally, administering the questionnaire every trimester may not have effectively identified factors influencing grit and QoL because the WHOQOL-BREF has been designed to evaluate QoL in the past 4 weeks. However, more frequent surveys could potentially lower participation rates and compromise data accuracy.

## Conclusion

Grit decreased throughout first-year residents’ internal medicine residency training. Grit was positively associated with QoL and SoLs and inversely associated with stress levels. Certain rotations were associated with lower grit, lower QoL, and higher stress. These findings may be helpful for further improvement of residency training.

## Electronic supplementary material

Below is the link to the electronic supplementary material.

**Supplementary Material 1: Additional file 1**. Appendix of additional information about questionnaires, including tools for evaluating grit, quality of life, stress level, and satisfaction of learning score, and Detail description of rotation in each training program

**Supplementary Material 2: Additional file 2**. Full forest plots of results

## Data Availability

Data is provided within the manuscript or supplementary information files.
