# Supplementary material for: Longitudinal study of association between quality of life and grit in internal medicine residents in Ramathibodi Hospital
Source: BMC Med Educ. 2024 Sep 30;24:1076. doi: 10.1186/s12909-024-06011-y (PMC11443821; doi:10.1186/s12909-024-06011-y)
Supplement: Supplementary file 1 — Supplementary Material 1: Additional file 1. Appendix of additional information about questionnaires, including tools for evaluating grit, quality of life, stress level, and satisfaction of learning score, and Detail description of rotation in each training program [file 12909_2024_6011_MOESM1_ESM.docx]

**Additional data 1**

List of questions in questionnaires.

1. Basic information

- What is your training year?
- Your assign ID
- Your training program.
- Age
- Gender
- Marital status (choice of Single, in relationship, Married)
- Your working experience (After graduation) (Answer as year)
- Habitat (Choice of private residence or Hospital dormitory)
- Financial status (Choice of Financial burden and non-financial burden

1. GRIT score

| **Question** | Very much like me | Mostly like me | Somewhat like me | Not much like me | Not like me at all |  |
| --- | --- | --- | --- | --- | --- | --- |
|  |  |  |  |  |  |  |
| 1. I often set a goal but later choose to pursue a different one. * |  |  |  |  |  |  |
| 2. I finish whatever I begin. |  |  |  |  |  |  |
| 3. Setbacks don’t discourage me. |  |  |  |  |  |  |
| 4. New ideas and projects sometimes distract me from previous ones* |  |  |  |  |  |  |
| 5. I have difficulty maintaining my focus on projects that take more than a few months to complete. * |  |  |  |  |  |  |
| 6. I become interested in new pursuits every few months* |  |  |  |  |  |  |
| 7. I am diligent |  |  |  |  |  |  |
| 8. I have overcome setbacks to conquer an important challenge. |  |  |  |  |  |  |
| 9. My interests change from year to year. * |  |  |  |  |  |  |
| 10. I have achieved a goal that took years of work. |  |  |  |  |  |  |
| 11. I have been obsessed with a certain idea or project for a short time but later lost interest. * |  |  |  |  |  |  |
| 12. I am a hard worker. |  |  |  |  |  |  |

Scoring:

1. For questions 2, 3, 7, 8, 10 and 12 assign the following points:

5 = Very much like me

4 = Mostly like me

3 = Somewhat like me

2 = Not much like me

1 = Not like me at all

2. For questions 1, 4, 5, 6, 9 and 11 assign the following points:

1 = Very much like me

2 = Mostly like me

3 = Somewhat like me

4 = Not much like me

5 = Not like me at all

2. Quality of life assessment tool: WHOQOL – BREF – THAI

What you think about your life in last four weeks.

|  | Never | Seldom | Quite often | Very often | Always |
| --- | --- | --- | --- | --- | --- |
| 1. How satisfied are you with your health? |  |  |  |  |  |
| 2. To what extent do you feel that physical pain prevents you from doing what you need to do? |  |  |  |  |  |
| 3. Do you have enough energy for everyday life? |  |  |  |  |  |
| 4. How satisfied are you with your sleep? |  |  |  |  |  |
| 5. How much do you enjoy life? |  |  |  |  |  |
| 6. How well are you able to concentrate? |  |  |  |  |  |
| 7. How satisfied are you with yourself? |  |  |  |  |  |
| 8. Are you able to accept your bodily appearance? |  |  |  |  |  |
| 9. How often do you have negative feelings such as blue mood, despair, anxiety, depression? |  |  |  |  |  |
| 10. How satisfied are you with your ability to perform your daily living activities? |  |  |  |  |  |
| 11. How much do you need any medical treatment to function in your daily life? |  |  |  |  |  |
| 12. How satisfied are you with your capacity for work? |  |  |  |  |  |
| 13. How satisfied are you with your personal relationships? |  |  |  |  |  |
| 14. How satisfied are you with the support you get from your friends? |  |  |  |  |  |
| 15. How safe do you feel in your daily life? |  |  |  |  |  |
| 16. How satisfied are you with the conditions of your living place? |  |  |  |  |  |
| 17. Have you enough money to meet your needs? |  |  |  |  |  |
| 18. How satisfied are you with your access to health services? |  |  |  |  |  |
| 19. How available to you is the information that you need in your day-to-day life? |  |  |  |  |  |
| 20. To what extent do you have the opportunity for leisure activities? |  |  |  |  |  |
| 21. How healthy is your physical environment? |  |  |  |  |  |
| 22. How well are you able to get around? |  |  |  |  |  |
| 23. To what extent do you feel your life to be meaningful? |  |  |  |  |  |
| 24. How satisfied are you with your transport? |  |  |  |  |  |
| 25. How satisfied are you with your sex life? |  |  |  |  |  |
| 26. How would you rate your quality of life? |  |  |  |  |  |

Scoring

Negative question 2,9,11. The rest are positive question.

Positive question score rating:

- Always = 5
- Very often = 4
- Quite often = 3
- Seldom = 2
- Never = 1

Negative question score rating:

- Always = 1
- Very often = 2
- Quite often = 3
- Seldom = 4
- Never = 5

Rating score for domain of Quality of life (QoL)

| Domain of QoL | Poor quality | Moderate quality | Good quality |
| --- | --- | --- | --- |
| Physical health | 7-16 | 17-26 | 27-35 |
| Psychological health | 6-14 | 15-22 | 23-30 |
| Relationship | 3-7 | 8-11 | 12-15 |
| Environment | 8-18 | 19-29 | 30-40 |
| Overall QoL | 26-60 | 61-95 | 96-130 |

The number of questions in each domain.

Physical health: number 2,3,4,10,11,12,24

Psychological health: number 5,6,7,8,9,23

Relationship: number 13,14,25

Environment: number: 15,16,17,18,19,20,21,22

Number 1 and 26 is in overall QoL, not in specific domain.

1. Stress evaluation: Srithanya-5 (ST-5)

| No. | Symptom or feeling in previous 2-4 weeks | Score | | | |
| --- | --- | --- | --- | --- | --- |
|  |  | 0 | 1 | 2 | 3 |
| 1 | Insomnia or hypersomnolence |  |  |  |  |
| 2 | Reduce focus |  |  |  |  |
| 3 | Anoying, Nervous, agitation |  |  |  |  |
| 4 | Boring, Bluemood |  |  |  |  |
| 5 | People avoidance |  |  |  |  |

ST-5 score interpretation.

0 – 4: Low stress

5 – 7: Moderate stress

8 – 9: High stress

10 – 15: Very high stress

5. Last three rotation you are working on

6. Satisfaction of learning score (SoLs) question

“How would you rate your satisfaction about your learning capabilities in last 3 months on the scale of 0-10.”

7. Any opinion on what you think affects your answer in this questionnaire?

8. The detail of each year training program

In the first year of training, residents undertake on-the-job training for general medicine rotations, including wards, intensive care units, outpatient units, and emergency room (ER). Additionally, residents have a 2-week vacation period. First-year residents do not undergo subspecialty rotation. Residents in the co-training program rotate to the co-training hospitals for 6 months. Residents in the main training program rotate to CNMI for 3 months.

In the second year of training, residents are rotated to subspecialty divisions, elective rotations, a 2-week vacation, and 3 months at a co-training hospital. Residents in the main program rotate to CNMI for 1 month and have 2 additional months of electives instead. In the final month of their training year, the main program residents take the rotation as the chief residents in the intensive care unit, intermediate ward, general ward, and ER. A board examination takes place 1 month before the end of the training year.

In third-year training, residents take responsibility as the chief residents in the intensive care unit, intermediate ward, general ward, and ER, as well as taking a 2-week vacation, electives, and 3 months at the co-training hospital. There are three elective rotations for the main program and five elective rotations for the co-training program. The last rotation for all third-year residents is an elective. A long case examination is conducted at the end of January, and the Assessment of Special Clinical Encounter exam [ASCE] is conducted at the end of the training year.

9. The table summarize the rotation in each training program
