# Supplementary material for: Longitudinal study of association between quality of life and grit in internal medicine residents in Ramathibodi Hospital
Source: BMC Med Educ. 2024 Sep 30;24:1076. doi: 10.1186/s12909-024-06011-y (PMC11443821; doi:10.1186/s12909-024-06011-y)
Supplement: Supplementary file 2 — Supplementary Material 2: Additional file 2. Full forest plots of results [file 12909_2024_6011_MOESM2_ESM.docx]

**Additional data 2**

Figures and tables


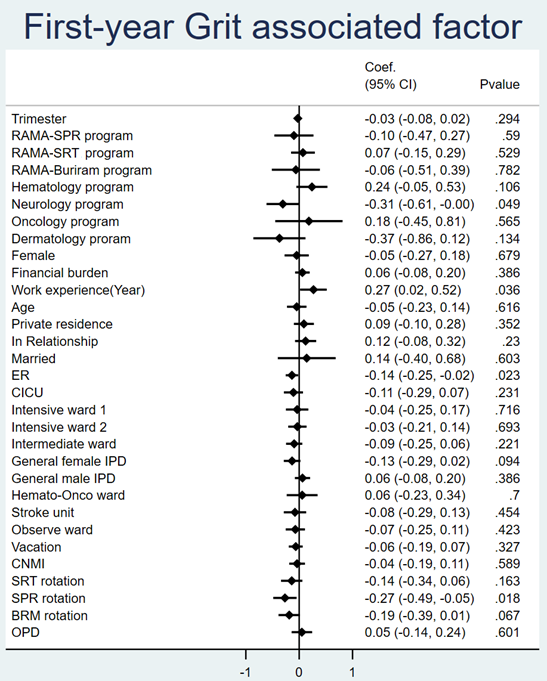


The analysis was controlled for training program, financial status, years of working experience, habitat, marital status, age, and rotation.

Figure 1: Forest plot of factors associated with grit in first-year residents.


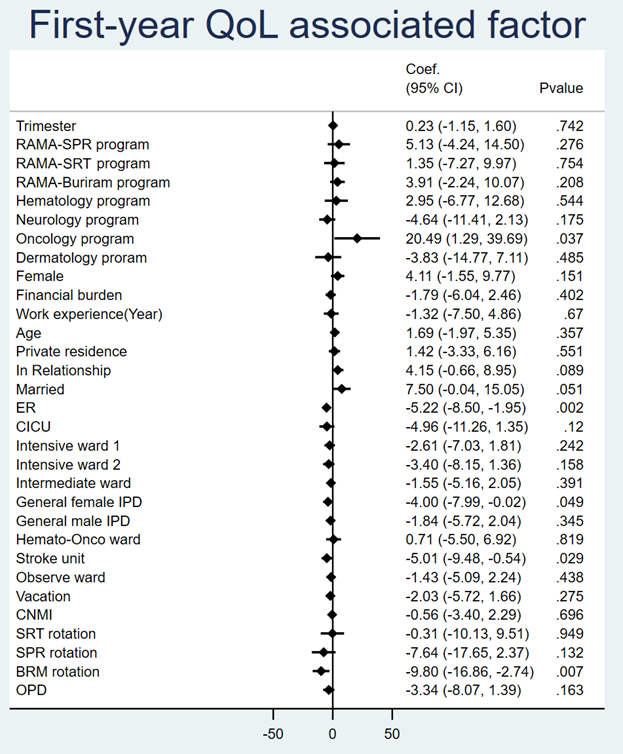


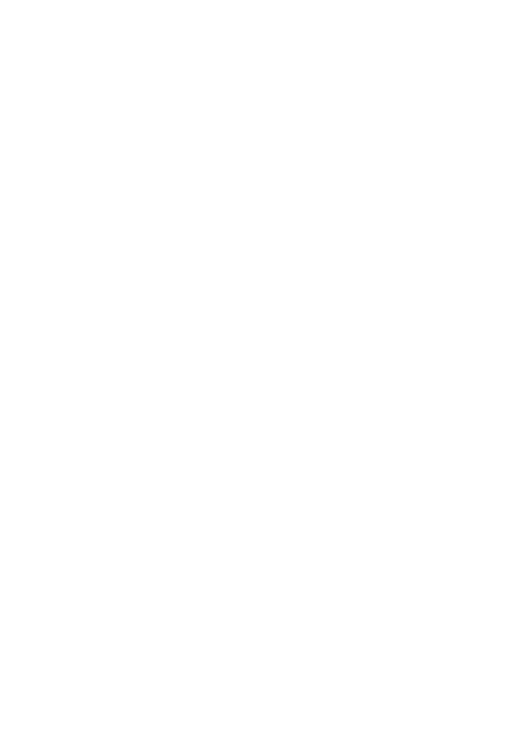

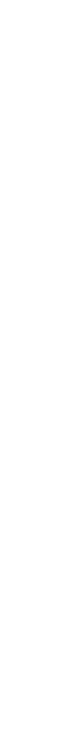


The analysis was controlled for training program, financial status, years of working experience, habitat, marital status, age, and rotation.

Figure 2: Forest plot of factors associated with QoL in first-year residents.


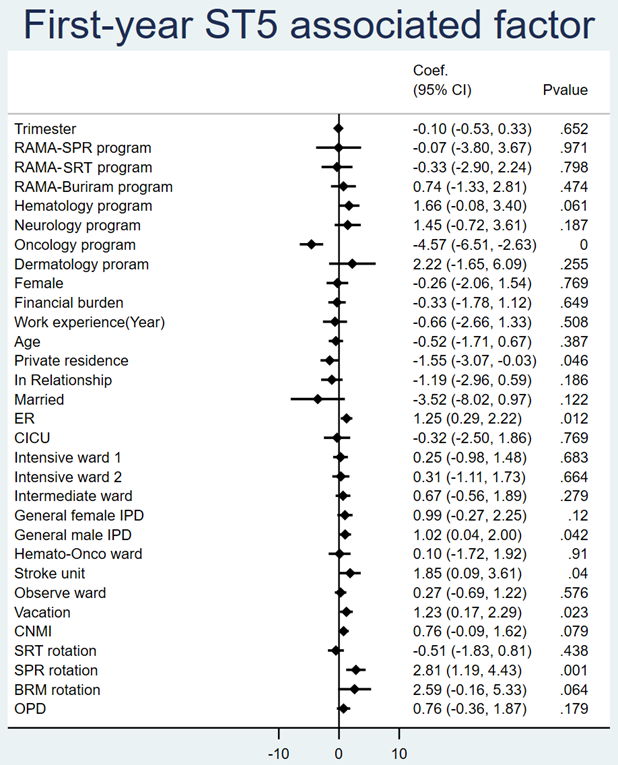


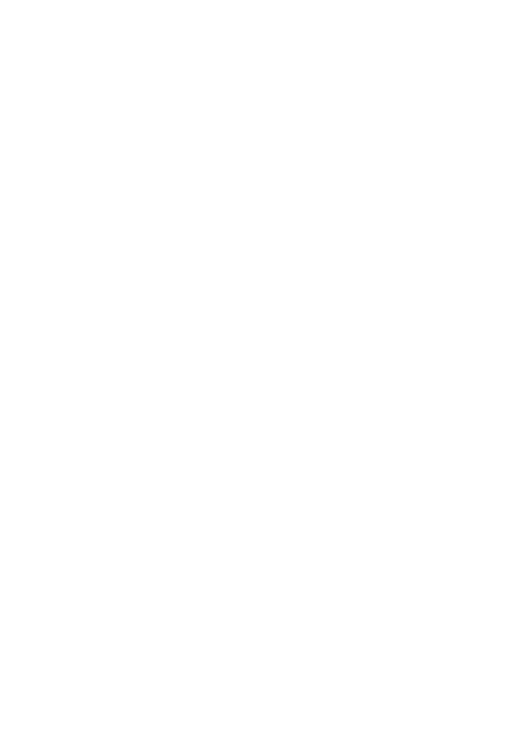

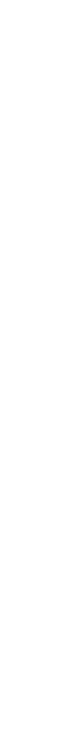


The analysis was controlled for training program, financial status, years of working experience, habitat, marital status, age, and rotation.

Figure 3: Forest plot of factors associated with ST-5 score in first-year residents.


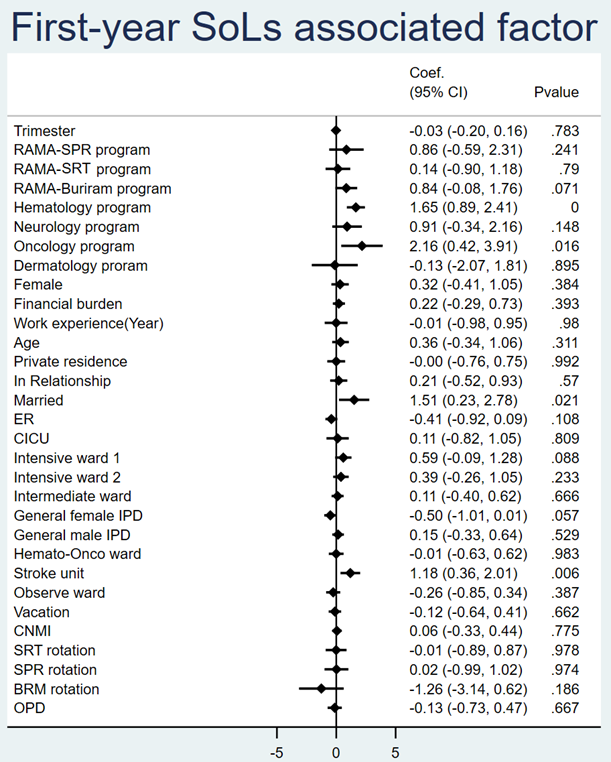


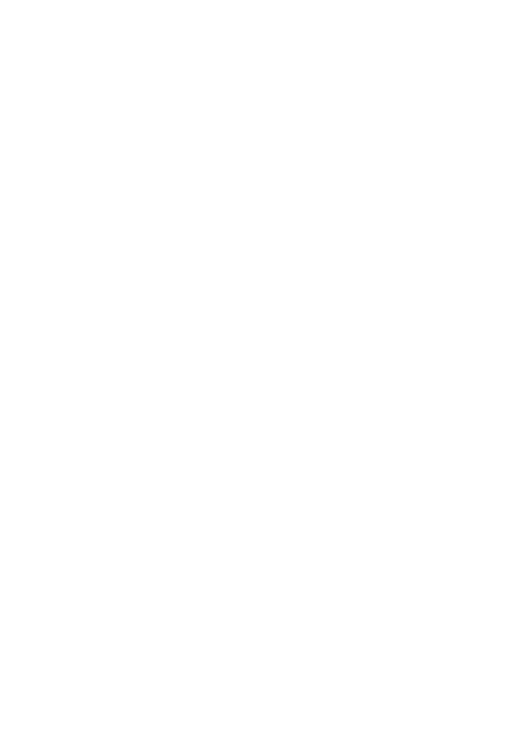

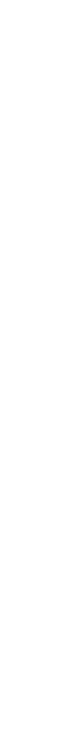


The analysis was controlled for training program, financial status, years of working experience, habitat, marital status, age, and rotation.

Figure 4: Forest plot of factors associated with satisfaction of learning score (SoLs) in first-year residents.


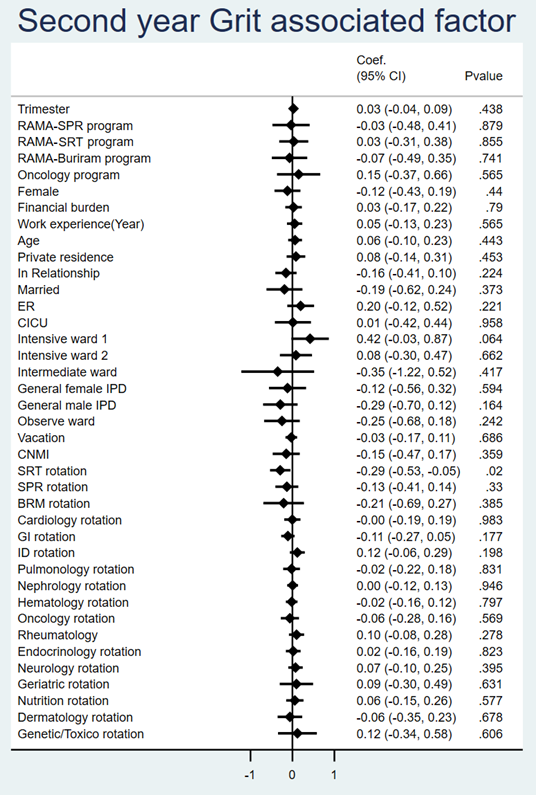


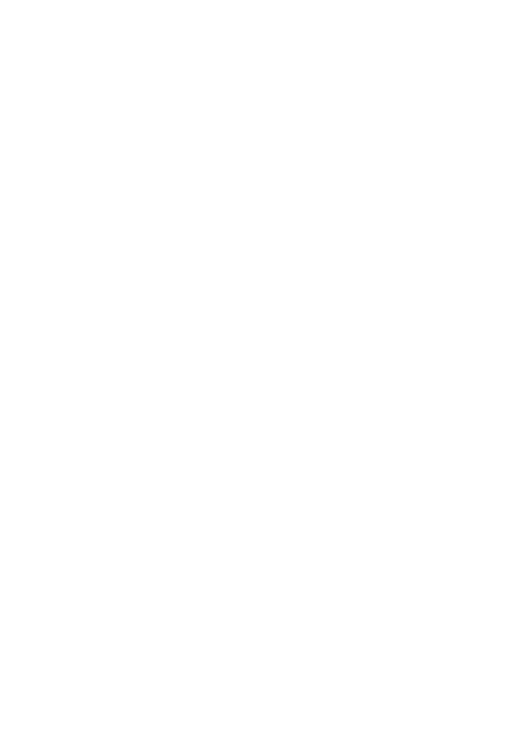

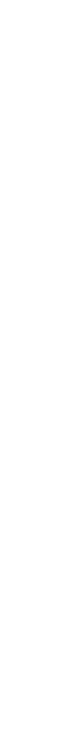


The analysis was controlled for training program, financial status, years of working experience, habitat, marital status, age, and rotation.

Figure 5: Forest plot of factors associated with grit in second-year residents.


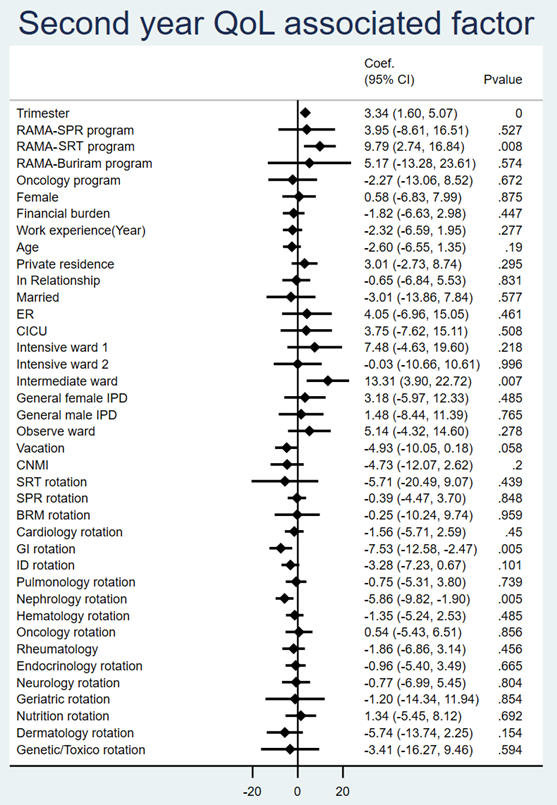


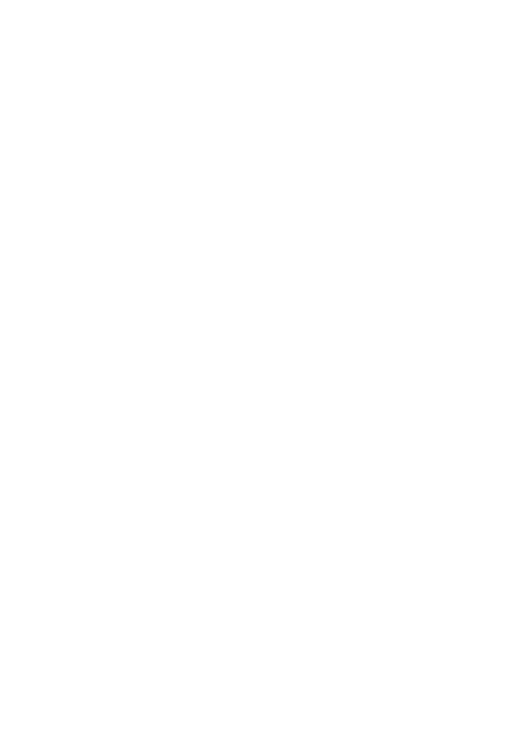

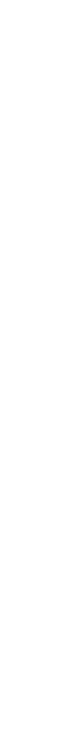


The analysis was controlled for training program, financial status, years of working experience, habitat, marital status, age, and rotation.

Figure 6: Forest plot of factors associated with QoL in second-year residents.


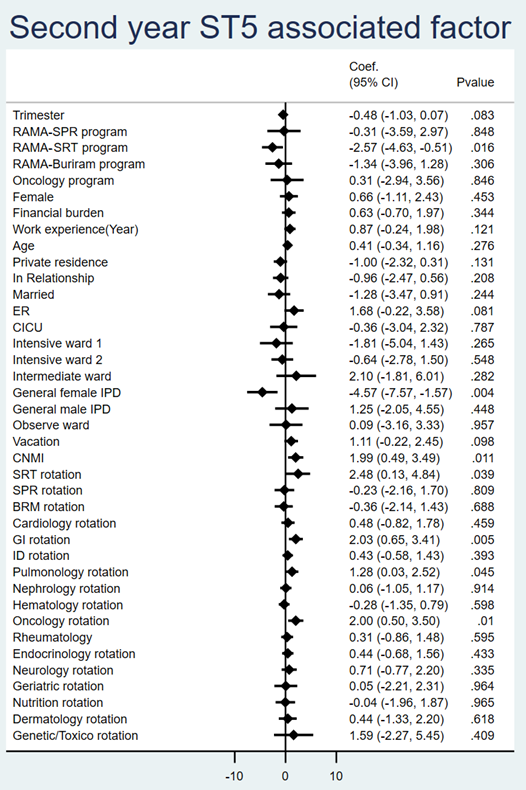


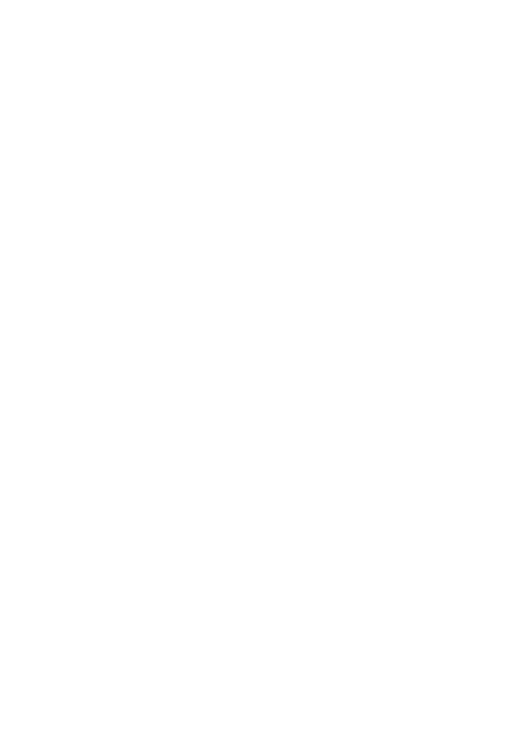

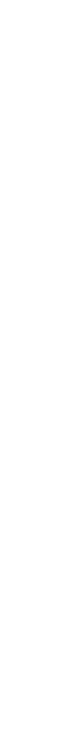


The analysis was controlled for training program, financial status, years of working experience,

habitat, marital status, age, and rotation.

Figure 7: Forest plot of factors associated with ST-5 score in second-year residents.


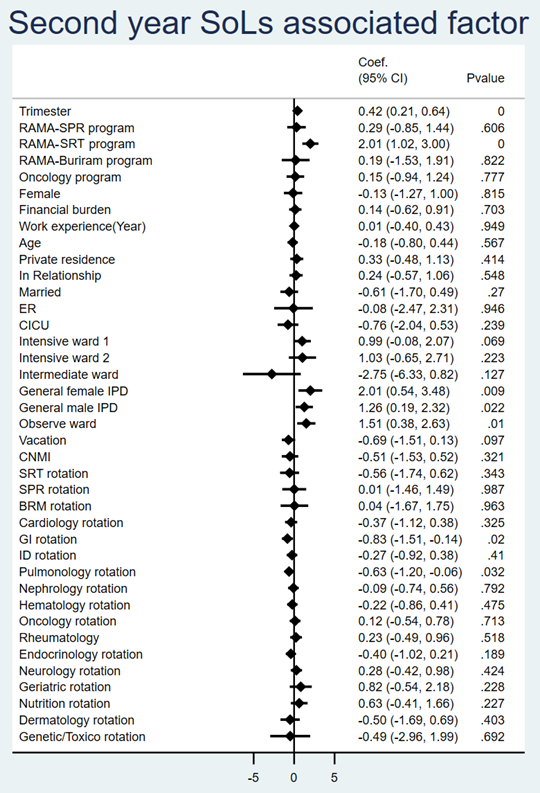


The analysis was controlled for training program, financial status, years of working experience, habitat, marital status, age, and rotation.

Figure 8: Forest plot of factors associated with satisfaction of learning score (SoLs) in second-year residents.


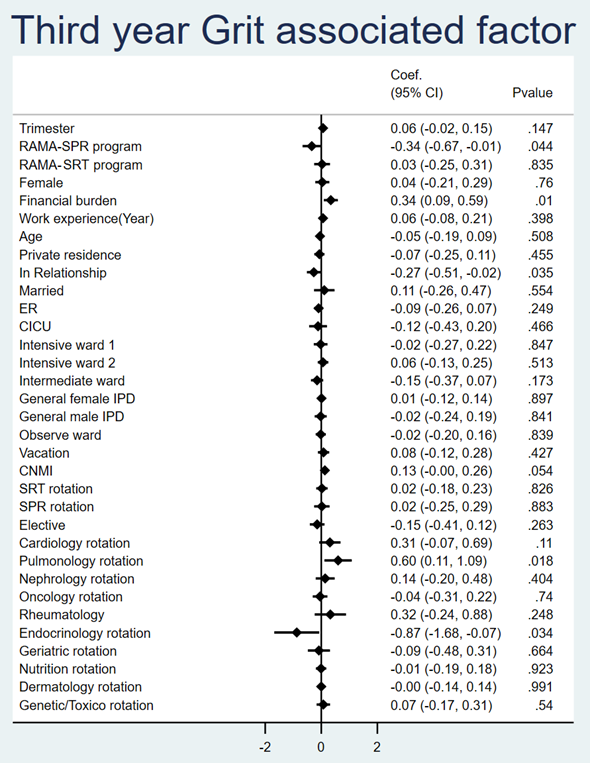


The analysis was controlled for training program, financial status, years of working experience, habitat, marital status, age, and rotation.

Figure 9: Forest plot of factors associated with grit in third-year residents.


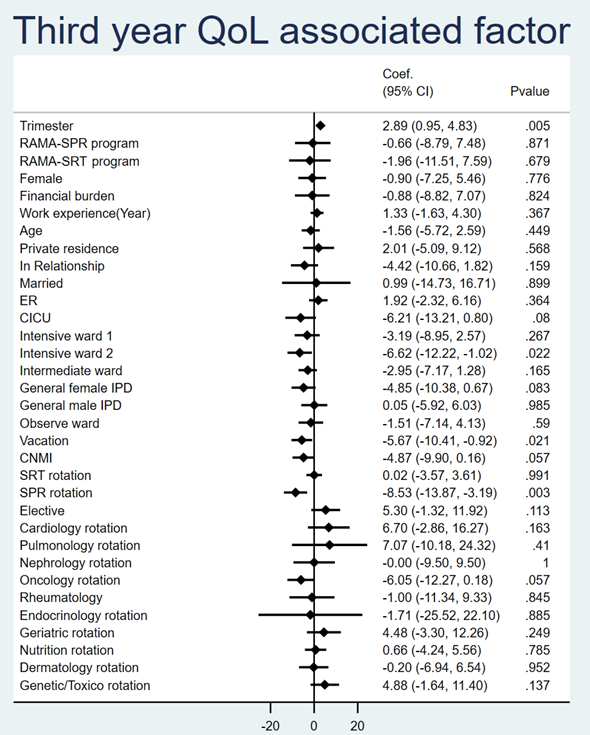


The analysis was controlled for training program, financial status, years of working experience, habitat, marital status, age, and rotation.

Figure 10: Forest plot of factors associated with grit in third-year residents.


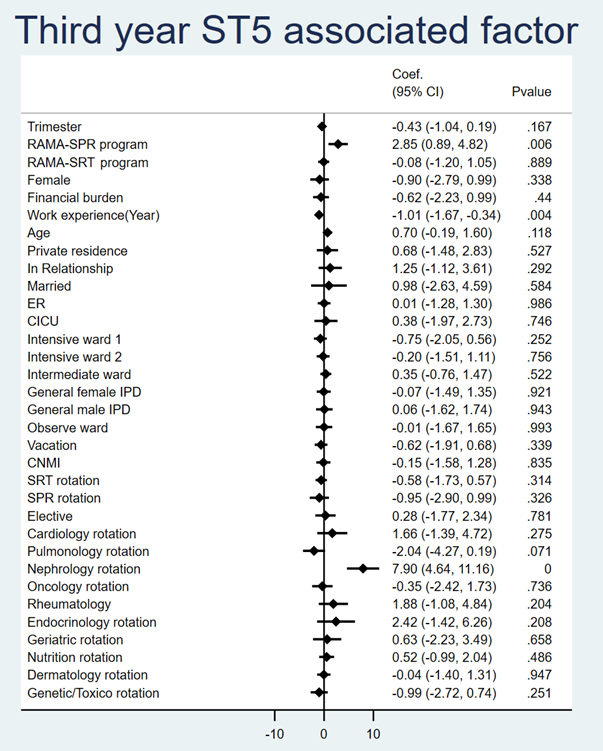


The analysis was controlled for training program, financial status, years of working experience, habitat, marital status, age, and rotation.

Figure 11: Forest plot of factors associated with ST-5 score in third-year residents.


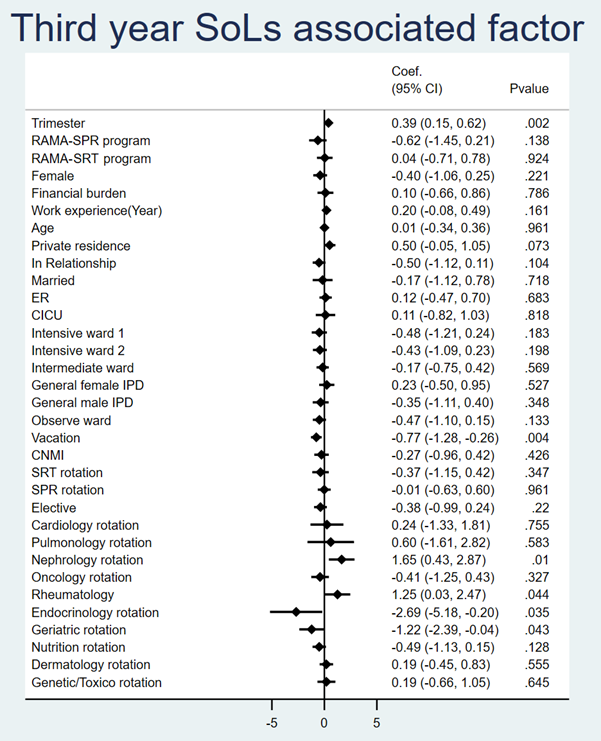


The analysis was controlled for training program, financial status, years of working experience, habitat, marital status, age, and rotation.

Figure 12: Forest plot of factors associated with satisfaction of learning score (SoLs) in third-year residents.
